# Effectiveness of community adolescent treatment supporters (CATS) interventions in improving linkage and retention in care, adherence to ART and psychosocial well-being: a randomised trial among adolescents living with HIV in rural Zimbabwe

**DOI:** 10.1186/s12889-019-6447-4

**Published:** 2019-01-28

**Authors:** Nicola Willis, Amos Milanzi, Mather Mawodzeke, Chengetai Dziwa, Alice Armstrong, Innocent Yekeye, Phangisile Mtshali, Victoria James

**Affiliations:** 1 Africaid, 12 Stone Ridge Way, Harare, Zimbabwe; 2 New Dimensions Consulting (NEDICO), 12 Stone Ridge Way, Harare, Zimbabwe; 3 International HIV/AIDS Alliance, Harare, Zimbabwe; 4 Independent Consultant, Harare, Zimbabwe; 5 Bristol Myers Squibb Foundation, Johannesburg, South Africa

**Keywords:** HIV, Adolescent, Peers, Adherence, Psychosocial wellbeing, Retention

## Abstract

**Background:**

Engagement with community adolescent treatment supporters (CATS) improves adherence, psychosocial well-being, linkage and retention in care among adolescents living with HIV. However, there is an urgent need for empirical evidence of the effectiveness of this approach, in order to inform further programmatic development, national and international policy, guidelines and service delivery for adolescents living with HIV. This study set out to determine the effectiveness of CATS services on improving linkage to services and retention in care, adherence and psychosocial well-being among adolescents living with HIV in Zimbabwe.

**Methods:**

A randomised trial was conducted in Gokwe South district, Zimbabwe over a period of 12 months. Ninety-four HIV-positive adolescents, 10–15 years old, on antiretroviral therapy were recruited to the study. 47 participants received standard of care from the Ministry of Health and Child Care and 47 received the same standard of care plus CATS services. Data collection involved a questionnaire which was administered at baseline then repeated at three, six, nine and twelve months for all participants. Survey questions on confidence, self-esteem and self-worth had a three-point Likert scale. Stigma, quality of life and the linkages to services and retention questions had a five-point Likert scale.

**Results:**

Survey questionnaires were completed with response rates of 40 out of 47 (85%) for the intervention arm, and 28 out of 47 (60%) for the control arm, at end-line. The intervention group were 3.9 times more likely to adhere to treatment compared to the control group. Linkage to services and retention in care within the intervention group increased compared with a decrease in the control arm. The intervention group reported a statistically significant increase in confidence, self-esteem, self-worth (*p* < 0.001) and quality of life compared (*p* = 0.028) with a decrease in the control arm.

**Conclusions:**

This study found that adolescents receiving the CATS service had improved linkage to services and retention in care, improved adherence and improved psychosocial well-being compared to adolescents who did not have access to such services.

**Trial registration:**

PACTR201711002755428. Registered 11 November 2017. Retrospectively registered.

## Background

There are an estimated 1.8 million adolescents living with HIV (ALHIV) worldwide, of which 82% live in sub-Saharan Africa [1]. This number is likely to increase due to improved survival of adolescents on antiretroviral therapy (ART) and the increasing number of new HIV infections among young people [2, 3]. Recent global efforts have re-focused attention on this age group, resulting in adolescent-focused guidance and implementation of HIV testing, treatment and care for adolescents [4, 5]. Zimbabwe, like other countries in the region, has scaled up initiatives to promote case finding, earlier diagnosis and ART initiation, resulting in an estimated 80% of ALHIV now on ART [6].

Despite this success of ART for adolescents, AIDS-related deaths among this age group are not declining and remain among the leading causes of death for this age group in sub-Saharan Africa [7, 8]. There is increasing evidence that suggests that ALHIV have poorer outcomes than children and adults across the HIV care cascade, including poorer retention in care, lower rates of virological suppression and higher rates of mortality [2, 9, 10]. Additionally, studies have indicated that ALHIV have an increased risk of poor mental health outcomes such as depression [11], which is itself associated with poor adherence [12]. Recognising these challenges for ALHIV, the 2016 WHO consolidated ART guidance now recommends that community-based interventions which support ART adherence and retention in care should also integrate psychosocial support and engage peers in service delivery [5, 13]. Several examples of group based interventions exist for ALHIV and there is some evidence that these have contributed to improved retention, psychosocial well-being and virological suppression [14–16]. However, there is a critical need for further evidence of the effectiveness of peer-led community services for ALHIV, particularly for those living in rural settings as most literature to date has focused on urban settings.

The Zvandiri programme is a model of differentiated service delivery for children, adolescents and young people, between the ages of 0 and 24 years, in Zimbabwe [17]. Adolescents and young people living with HIV between the ages of 18 and 24 years are trained and mentored by Africaid and the Ministry of Health and Child Care (MoHCC) as peer counsellors. These peer counsellors are known as Community Adolescent Treatment Supporters (CATS). After training, the CATS deliver adherence and psychosocial support in their own communities – in health facilities and the homes of other HIV positive children, adolescents and young people. Some programmatic data from Harare, Zimbabwe, suggest that engagement with a CATS improves linkage to services, retention care, adherence and psychosocial well-being and retention in care among young people living with HIV. No previous studies have been conducted on this topic in rural Zimbabwe. However, there is an urgent need for more empirical evidence of the effectiveness of this approach, in order to inform further programme development, national and international policy, guidelines and service delivery for ALHIV.

This study set out to determine the effectiveness of CATS services on improving linkage to services and retention in care, adherence and psychosocial well-being among 100 ALHIV in a rural district of Zimbabwe.

## Methods

### Study design

**Fig. 1:**
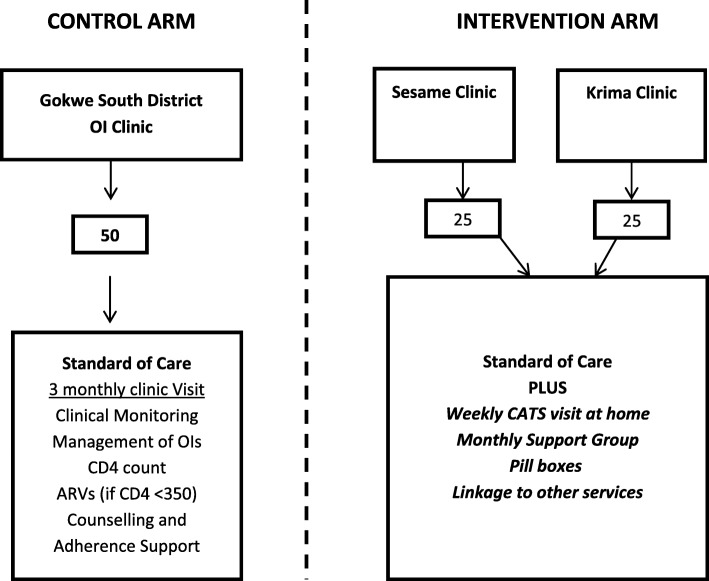
Study Design. The study used a randomised trial research design. Forty-seven adolescents living with HIV were assigned to the control arm and received standard of care; 47 were assigned to the intervention arm (Krima and Sesame clinic) and 47 received standard of care with the addition of CATS support (Gokwe South Hospital)

Between December 2014 and November 2015, a 12-month longitudinal survey was conducted in Gokwe South, a rural district in Midlands province, Zimbabwe. Gokwe South District is a communal farming, informal mining community. The study used a randomised trial research design (Fig. 1). Three study sites were randomly selected in consultation with the MoHCC, including two clinics in the intervention arm and one larger clinic in the control arm.

### Study participants

Ninety-four ALHIV, 10–15 years old, on ART, were recruited to the study from the three study sites (2 intervention and one control). Random number generation in Microsoft Excel was used to generate the random allocation sequence, with 47 participants allocated in each arm. The study used blocked randomisation with a block size of 10 which is ideal given the small sample size. The Ministry of Health and Child Care with technical assistance from Bristol Myers-Squibb generated the random allocation sequence used to assign participants to the control and intervention arm and enrolled the participants to the study. All participants were on ART, aware of their HIV status and not involved with other support services. Participants were all receiving their HIV treatment and care at the study sites prior to the start of the study. At each study site, eligible participants were informed of the study during their routine clinic visit and invited to participate in the study.

### Standard of care

Participants in the control arm received the standard of care provided by the MoHCC, including monthly clinic reviews, ART, adherence counselling, CD4 monitoring and management of opportunistic infections. Treatment and care was led by a nurse and/or a primary counsellor.

### Intervention

Participants in the intervention arm received the same standard of care, but were also allocated to one of nine trained and mentored CATS for additional support. This included a weekly home visit during which the allocated CATS provided HIV and ART information and counselling as well as monitored the participants’ adherence and general well-being. In the event that the participant was unwell or faced difficulties with adherence, the CATS would refer the participant to the CATS mentor in their district. The mentor would then liaise with the participants’ clinic for follow-up. CATS additionally supported caregivers with information and counselling. Participants requiring referral for other services, such as social welfare, were identified through support group or home visits, and were referred accordingly. All nine CATS attended a weekly feedback meeting with the CATS Mentor at the clinic. Participants in the intervention arm were also encouraged to attend a support group, if they wished.

### Data collection

The study utilised a questionnaire to collect quantitative data on the impact of the CATS intervention on self-reported adherence, psychosocial well-being and retention in care. The survey questionnaire was developed from previously validated questionnaires used in an earlier study among adolescents living with HIV enrolled in the Zvandiri programme [12]. Data collection involved a questionnaire administered at baseline, which was then repeated at three, six, nine and twelve months for all participants, including those in the intervention and control arms.

### Questionnaire

The questionnaire was comprised of five components, namely: demographic information; adherence; psychological well-being, and linkages and retention in care. The questionnaire was translated into Shona, the local language. Data was collected using printed questionnaires. There were 71 closed-ended questions on the survey, i.e. 9 questions on demographics, 16 questions on adherence, 36 questions on psychological well-being and 10 questions on linkage and retention in care. The first 11 adherence questions were on knowledge which used “2 = Yes” for those with adherence knowledge, “1 = a little”, “0 = No” for those without adherence knowledge and “99 = not applicable”. The last five questions on adherence, used “1 = Yes” and “0 = No”. The mental health sections had three sections namely; (i) confidence, self-esteem and self-worth and had a three-point Likert scale: “2 = Yes”, “1 = a little” and “0 = disagree” (ii) stigma and (iii) quality of life sections had a five-point Likert scale: “4 = strongly agree”, “3 = agree”, “2 = neutral”, “1 = disagree” and “0= strongly disagree”. The linkages to services and retention questions were answered using five options, “3 = very much”, “2=a moderate amount”, “1= a little” and “0=not at all”.

The questionnaire was pretested with 10 respondents in Gokwe in a separate health centre. Pre-testing assessed the understanding of procedures for administering questionnaires among adolescents living with HIV, as well as the validity and reliability of questions. As a result, some questions were simplified or omitted to prevent response bias.

Trained enumerators conducted data collection. The training familiarised the enumerators with the Zvandiri programme, background and justification for this study, objectives of the study, study methodology, ethical considerations and general research knowledge.

### Data analysis

Data from the baseline, quarterly and twelve-month surveys was analysed using descriptive statistics (proportions, means or point system with 4 being highest while 0 was least) and odds ratios to provide evidence of effectiveness of the CATS intervention on improving linkages and retention, adherence and psychosocial well-being and linkages to health retention and care.

### Ethical considerations

Ethics approval was granted by the Medical Research Council of Zimbabwe in 2014. Written informed consent was obtained from the caregivers of all participants, and participants were required to give assent prior to their participation in the study.

## Results

### Demographic data

**Fig. 2:**
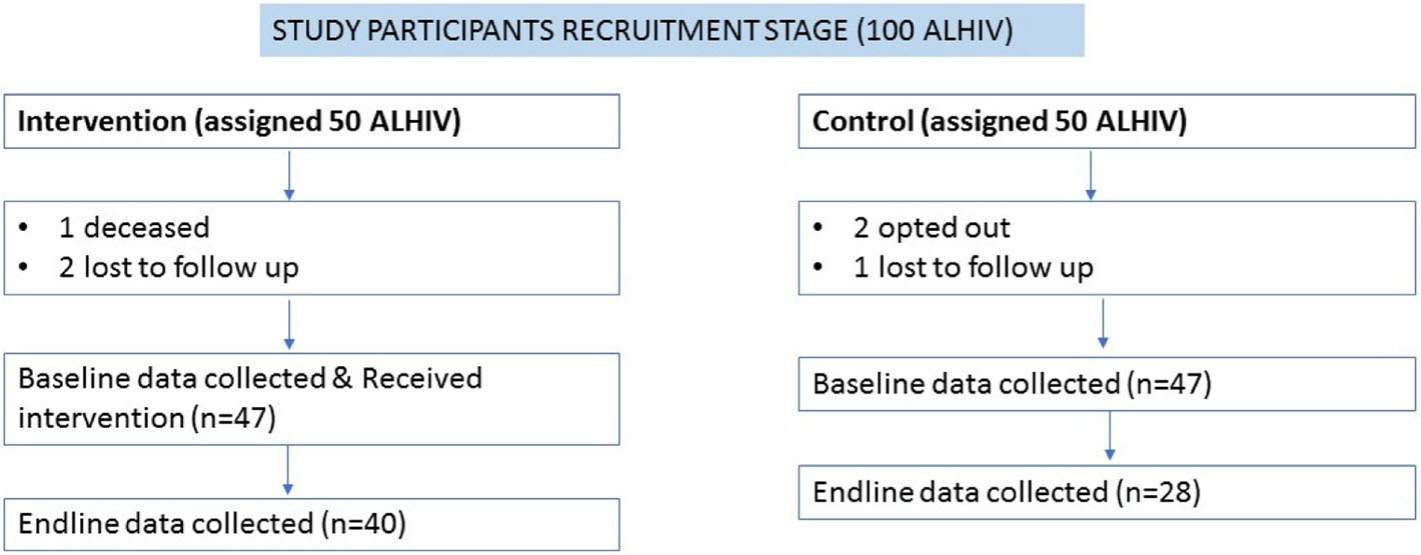
Consort diagram

One hundred participants were recruited in to the study, with 50 participants in each arm. However, one participant died and two were lost to follow up in the intervention arm; two participants opted out and one was lost to follow up in the control arm. A total of 47 participants completed the study in each arm (Fig. 2).

The majority of participants were female in both intervention (60%) and control arms (62%) at baseline. Most participants in the intervention arm were aged 10 years (33%) followed by those aged 13 (19%) and 14 years (19%). Orphanhood was common with 46% (intervention arm) and 33% (control arm) of participants having lost both parents. Less than 10% were maternal orphans while 17% were paternal orphans. Nearly all respondents were enrolled in primary school education in both the intervention (96%) and control arm (98%). Most ALHIV were living in large household sizes with 4–7 members (46% intervention arm and 64% in control arm).

Survey questionnaires were completed with response rates of 40 out of 47 (85%) for the intervention arm, and 28 out of 47 (60%) for the control arm, at end line (Table 1).

**Table 1:** Demographic characteristics of respondents

Demographic Characteristic	Baseline (% of total)		End line (% of total)	
	Intervention	Control	Intervention	Control
Ward				
Chisina/Krima/Chisina 3	50.0	2.1	45.0	0.0
Nemangwe/Nemangwe 3	50–0	0.0	50.0	19.2
Njelele	0.0	97.9	5.0	80.8
Age Group				
10	32.7	17.0	12.5	7.1
11	5.8	8.5	12.5	14.3
12	9.6	14.9	12.5	10.7
13	19.2	12.8	12.5	17.9
14	19.2	21.3	15.0	25.0
15	13.5	25.5	25.0	17.9
16	0.0	0.0	10.0	7.1
Sex				
Male	40.4	38.3	47.5	39.3
Female	59.6	61.7	52.5	60.7
Parental Status				
Both parents are alive	26.9	40.4	20.0	11.1
Mother alive, father dead	17.3	17.0	25.0	33.3
Father alive, mother dead	7.7	14.9	22.5	22.2
Both parents dead	46.2	27.7	32.5	33.3
Not known	1.9	0.0	0.0	0.0
Currently in School				
Yes	96.2	97.9	92.5	96.4
No	3.8	2.1	7.5	3.6
Level at School				
Primary school	86.0	73.9	78.4	66.7
Secondary School	14.0	26.1	21.6	29.6
High School	0.0	0.0	0.0	3.7
Currently not in School (due to)				
Completed Ordinary /Advanced Level	0.0	100.0	0.0	0.0
Did not have money for school fees	100.0	0.0	66.7	0.0
Dropped out because of ill health	0.0	0.0	33.3	100.0
Number of People per household				
1–3	36.5	23.4	15.0	17.9
4–7	46.2	63.8	60.0	67.9
8–10	13.5	10.6	25.0	10.7
11+	3.8	2.1	0.0	3.6
N	47	40	47	28

Demographic characteristics of respondents, including ward, age cohort, sex, parental status, schooling status and level, reasons for not attending school and size of household. Demographic characteristic are given for both the intervention and control arm, and both at baseline and endline. *N* = 47 for the intervention arm at baseline, *N* = 40 for the control arm at baseline, *N* = 47 for the intervention arm at end line, and *N* = 28 for the control arm at endline

### Linkage to services and retention in care

The intervention group reported a statistically significant improvement in both linkage to services and retention in care between baseline and end line (Table 2). Linkage to services within the intervention group increased from 2.38 points to 3.38 points (*p* < 0.001) while retention to care increased from 3.66 to 3.74 (*p* < 0.001). Though lower increases were noted in the control group, there was also a statistically significant increase in linkage to healthcare (*p* < 0.001). However, retention in care within the control group declined from 3.86 points at baseline to 3.31 points at end line.

**Table 2:** Linkage and Retention in care

	Intervention				Test for significance	Control				Test for significance
	Baseline		End line		*P*-value	Baseline		Endline		*P*-value
	Mean score(95%CI)	Std Dev	Mean score(95%CI)	Std Dev		Mean score(95%CI)	Std Dev	Mean score(95%CI)	Std Dev	
Linkages	2.38 (2.21;2.54)	0.0824	3.38 (3.20;3.56)	*0.0897*	**< 0.001**	2.34 (2.23;2.45)	*0.0558*	2.69 (2.46;2.91)	*0.1136*	**< 0.001**
Retention	3.66 (3.54;3.79)	0.0633	3.74 (3.62;3.86)	*0.0620*	**< 0.001**	3.86 (3.79;3.92)	*0.0326*	3.31 (3.13;3.50)	*0.0941*	**< 0.001**
N	47		47			40		28		

Linkage and Retention in care, is measured using mean score (including 95% CI) given for both the intervention and control arm, and both at baseline and endline. N = 47 for the intervention arm at baseline, N = 40 for the control arm at baseline, *N* = 47 for the intervention arm at end line, and N = 28 for the control arm at endline. Difference between baseline and endline is statistically significant if *p* < 0.05. The **bold text** under *p*-value column show statistical significance

In the intervention arm, there was an improvement in the number of adolescents who are satisfied with the referrals that have been made for them (23% at baseline to 45% at end line) (*p* = 0.026) and the connection with other helpful young people who are friendly and could understand their situation (10% at baseline to 88% at end line). However, many of these adolescents felt their community does not support them in accessing quality treatment and care (50% at baseline to 23% at end line) (*p* = 0.007). Adolescents felt supported by CATS and support group members in getting the right care and treatment when they needed it (69% at baseline to 93% at end line) (*p* = 0.006). Yet adolescents reported that they were less comfortable discussing their health concerns at local clinics (89% at baseline to 85% at end line) (*p* = 0.571), less satisfied with the medical advice they received from their local clinics (87% at baseline to 78% at end line) (*p* = 0.256) and a lower proportion felt doctors and nurses at their local clinics were accessible when they needed them (75% at baseline to 63% at end line) (*p* = 0.217).

### Adherence

Participants receiving the intervention reported a statistically significant improvement in adherence to ART from 44.2% at baseline to 71.8% at end line (*p*-value = 0.008). The intervention group were 3.9 times more likely to adhere to treatment compared to the control group (OR = 3.934) (Table 3).

**Table 3:** Percentage of adolescents adhering to ART, before and after CATS intervention

Research Arm	Baseline (%)	Count (N)	End line (%)	Count (N)	Difference (95% CI)	*P* value	Odds Ratio (95% CI)
Intervention	44.2%	47	71.8%	47	27.6 (7.0, 48.2%)	**0.0087**	3.934 (1.404, 11.02)
Control	48.9%	39	39.3%	28	9.6% (−13.7, 32.9%)	0.419	

Percentage of adolescents adhering to ART, before and after CATS intervention is given for both the intervention and control arm, and both at baseline and endline. N = 47 for the intervention arm at baseline, *N* = 39 for the control arm at baseline, *N* = 47 for the intervention arm at end line, and *N* = 28 for the control arm at endline. Difference between the baseline and endline is provided (including the 95% CI). Difference between baseline and endline is statistically significant if *p* < 0.05. The **bold text** under *p*-value column show statistical significance. Odd ratios are further used for statistically significant differences

### Psychosocial well-being

The intervention group reported a statistically significant increase in confidence, self-esteem and self-worth by 0.49 points (*p* < 0.001). The intervention group reported a decline in stigma, though it was not statistically significant (*p* = 0.848). The control group, however, experienced a statistically significant increase in stigma levels (*p* = 0.01). The intervention group reported a statistically significant improvement in the quality of life by 0.29 points (*p* = 0.028). The control group experienced a statistically significant decline in the quality of life by 0.26 points. (Table 4, Table 5, Table 6).

**Table 4:** Mean respondents’ confidence, self-esteem and well-being scores, before and after CATS intervention

Research Arm	Baseline (Mean Score)	Count (N)	End line (Mean Score)	Count (N)	Difference (95% CI)	*P* value
Intervention	2.21	47	2.70	47	0.49 (0.313,0.667)	**< 0.001**
Control	2.45	39	2.60	28	0.15 (−0.018,0.318)	0.078

Mean respondents’ confidence, self-esteem and well-being scores, before and after CATS intervention is measured using mean score given for both the intervention and control arm, and both at baseline and endline. N = 47 for the intervention arm at baseline, N = 39 for the control arm at baseline, N = 47 for the intervention arm at end line, and N = 28 for the control arm at endline. Difference between the baseline and endline is provided (including the 95% CI). Difference between baseline and endline is statistically significant if *p* < 0.05. The **bold text** under *p*-value column show statistical significance

**Table 5:** Mean respondents’ stigma scores, before and after CATS intervention

Research Arm	Baseline (mean point count)	Count (N)	End line (mean point count)	Count (N)	Difference (95% CI)	*P* value
Intervention	2.79	37	2.77	24	0.02% (− 0.188, 0.228%)	0.848
Control	2.52	39	3.03	17	0.51% (0.13, 0.89%)	**0.01**

Mean respondents’ stigma scores, before and after CATS intervention is measured using mean score given for both the intervention and control arm, and both at baseline and endline. *N* = 37 for the intervention arm at baseline, N = 39 for the control arm at baseline, *N* = 24 for the intervention arm at end line, and *N* = 17 for the control arm at endline. Difference between the baseline and endline is provided (including the 95% CI). Difference between baseline and endline is statistically significant if *p* < 0.05. The **bold text** under *p*-value column show statistical significance

**Table 6:** Mean respondents’ quality of life scores, before and after CATS intervention

Research Arm	Baseline (mean point count)	Count (N)	End line (mean point count)	Count (N)	Difference (95% CI)	*P* value
Intervention	**3.16**	47	**3.450**	47	0.29 (0.031,0.549)	**0.028**
Control	**3.61**	39	**3.35**	28	0.26 (0.61,0.459)	**0.011**

Mean respondents’ quality of life scores, before and after CATS intervention is given for both the intervention and control arm, and both at baseline and endline. N = 47 for the intervention arm at baseline, N = 39 for the control arm at baseline, *N* = 47 for the intervention arm at end line, and N = 28 for the control arm at endline. Difference between the baseline and endline is provided (including the 95% CI). Difference between baseline and endline is statistically significant if *p* < 0.05. The **bold text** under *p*-value column show statistical significance

## Discussion

This study set out to determine the effectiveness of Community Adolescent Treatment Supporters (CATS) services on improving linkage to services and retention in care, adherence and psychosocial well-being among 10 to 15-year old ALHIV in a rural district of Zimbabwe. Our findings showed that adolescents receiving CATS services had improved linkage to services, retention in care, self-reported adherence and psychosocial well-being compared to adolescents who did not have access to CATS services.

Engagement with health care services ensures that adolescents with HIV are provided the medical care they need, treatments including ART, information, skills and support. It also gives them the opportunity to express their concerns, highlight needs they may have and learn from others. The goal of these services is to improve the health and well-being of individual adolescents. Yet, adolescents with HIV have been found to have poorer retention in care compared with adults [2]. This is likely due to a variety of individual, social and system related issues. Individual issues include competing priorities, such as: school, social and family commitments, busy routines and limited understanding of the need for engaging in services [4]. Social issues include lack of support or neglect at home, changing households and lack of financial resources for transport or services. At the clinical level, services may not be available at times which are convenient for adolescents, service providers may lack the skills to provide adolescent-responsive services [4, 9].

The results of this study show that adolescents receiving CATS services had improved linkage to services and retention in care. CATS are well placed to assist their peers with information about services and to assist them in accessing those services, and to help them remain engaged in care. Differentiated care models of care are now being rolled out in different countries and aim to reduce the frequency of clinic visits for individuals who are virologically suppressed [13]. Yet the clinical and psychosocial circumstances of adolescents are constantly evolving. Community, peer-led interventions should therefore be a critical component of differentiated service delivery for adolescents in order to ensure active follow up and tracking of individual adolescents [18]. As young people living in the community, CATS are able to reach young people at their point of need in a confidential, safe manner and ensure that they are then linked to the services that they need.

Early initiation of ART is now known to improve mortality and morbidity among people living with HIV, including adolescents [3]. In response, the 2016 WHO ART guidelines recommended early initiation of ART for all people diagnosed with HIV [5]. Yet the success of ART requires optimal adherence to antiretroviral medication [10]. One recent baseline study in Zimbabwe found that, in their cohort, 48% of ALHIV on ART had virologic failure (viral load ≥1000 copies/ml) [18]. As the number of adolescents on long term ART continues to increase, as well as the number of those newly initiated on ART, it is critical that national ART programmes be accompanied with evidence-based interventions which have been found to support adherence, so that adolescents are not only initiated on ART successfully, but that they continue to adhere. Adolescents newly initiating treatment may still be coming to terms with their HIV status, may not have a treatment supporter and may have many fears around commencing treatment. Those already on treatment may experience a range of difficulties with adherence, including forgetting to take their medications, pill fatigue, lack of adherence support, or they may be concealing their medication due to fears of stigma and discrimination. Factors contributing to improved adherence in adolescents include treatment literacy, treatment reminders, coping strategies and support from a family member or friend [4]. Peers are well placed to provide this due to their shared experiences and understanding. Similarly, CATS are well placed to provide ART information in a way which other children and adolescents can understand and relate to; they are able to identify and relate with the various challenges which their peers experience and draw on their own experiences to equip them with coping skills and motivation to adhere.

There is increasing evidence that adolescents with HIV are at risk of common mental health disorders [11, 12, 19] and that this affects adherence to ART [12, 20], as a result of the various range of psychosocial stressors in their lives. This includes orphaning, grief, stigma and discrimination, coping with their HIV diagnosis, fears for their future and mortality. A recent study in Zimbabwe found that adolescents with HIV and depression attributed their poor mental health to their negative relationships with families and peers [20]. They identified supportive relationships with family members and peers as being central to the support they required. In this study, adolescents receiving CATS services had improved confidence, self-esteem and self-worth, compared with adolescents not receiving CATS services. WHO now recommends the integration of mental health within HIV service delivery for all age groups [5]. There is strong evidence to suggest that lay cadres are effective in providing mental health services for people living with HIV in the adult population [21]. While this approach may also be applied to CATS as lay mental health providers [22], there is no empirical evidence to support this yet. Further investigation of the role of CATS in providing mental health services is now needed.

The findings from this study have contributed to the adoption of the CATS service by the Ministry of Health and Child Care in Zimbabwe as a model of differentiated service delivery for children, adolescents and young people. This intervention is being scaled up across the country, alongside other models of differentiated care. The CATS model has also been documented in the 2017 guidance on differentiated service delivery for adolescents and young key populations [18]. There is now need for further evaluation of this intervention at scale to measure its effectiveness and replicability and to ensure quality mentorship support and supervision is in place for this cadre of young people.

### Limitations

A potential limitation of this study is that the sample size was small and it may therefore not be possible to make generalisations about the larger population of ALHIV in Gokwe South district, nor other districts in Zimbabwe. In addition, there was a relatively low response rate at end line in the control arm as participants in this arm were not routinely attending clinic as scheduled. Furthermore, as viral load monitoring was unavailable, this study focused on self-reported adherence, which may be unreliable. Two larger randomised control trials are currently underway in Zimbabwe and address these limitations.

## Conclusion

Data from this study suggest that adolescents living with HIV receiving the CATS service have improved linkage and retention in care, improved adherence and improved psychosocial well-being, compared with adolescents who do not have access to CATS service. Importantly, unexpected outcomes also emerged from the study, including improvements in disclosure rates from caregivers to their children and strong support for the use of pill boxes and adolescent-friendly counselling tools. Furthermore, CATS were accepted and valued by caregivers and health workers. This evidence has been utilised to inform the scale-up of CATS services within the district, as well as other districts across Zimbabwe with the support of the Ministry of Health and Child Care. Further research is now needed to establish the effectiveness of the CATS service on a larger scale and on viral suppression to provide additional evidence around the effectiveness of this model.

## Abbreviations

AIDS: Acquired immune deficiency syndrome
ALHIV: Adolescents living with HIV
ART: Antiretroviral therapy
ARVs: Antiretroviral medicines
CATS: Community adolescent treatment supporters
HIV: Human immunodeficiency virus
MoHCC: Ministry of health and child care
WHO: World health organisation
